# Cotton-seed Disease1Read at the meeting of the Missouri Valley Veterinary Association, June, 1899.

**Published:** 1899-09

**Authors:** F. C. McCurdy

**Affiliations:** Kansas City, Mo.


### COTTON-SEED DISEASE.[1]

By Dr. F. C. McCurdy,
KANSAS CITY, MO.

Under the name of "Cotton-seed Disease" this paper was prepared for the purpose of describing the acute symptoms seen in cattle that have been overfed on a concentrated and exclusive diet of cotton-seed, and also a well-known lesion from the same cause that has been called "cotton-seed blindness."

During last April, while a herd of small Southern cattle was passing through the stock-yards it was observed that all had sore eyes—that many seemed partially blind, and in a few there was a

[1] Read at the meeting of the Missouri Valley Veterinary Association, June, 1899.

complete loss of sight. At first glance it was supposed that they were suffering with contagious ophthalmia. As they were being driven it was noticed that they were very nervous, weak, and exhausted. They moved with an uncertain, staggering gait. Respiration seemed hurried and difficult, and there was a frequent "scouring" discharge from the bowels.

On reaching the pens they showed a condition of nervous depression, remaining in one position, either recumbent or standing with the head down, with trembling of the limbs and without ruminating. The owner said that their poor condition was caused quite recently by his mistake of feeding them too much cotton-seed.

One steer, about the size of a native yearling, had been separated and tagged as a "downer." It was in great distress, and was indeed an exceptionally acute and fatal case. This animal was lying on the side, with the head extended, and was making convulsive movements of the limbs, at intervals trying to rise. When approached he became very nervous and excited, making a great effort to gain his feet, but failing he immediately plunged forward and remained prostrate, being too weak to make a further struggle. With the head extended, mouth open, the tongue protruding, and of a blue color, the respiration being hurried and stertorous, this animal seemed in danger of suffocating. It was noticed in this steer there was a jugular venous pulse, irregular in rate, but continuous, and varying in quantity. The eyes had small opaque areas in the corners, and the temperature taken in the rectum was over 109° F. This animal was slaughtered a few hours later, and the carcass was condemned by a meat-inspector because of extensive bruises, and, unfortunately, as the animal had been previously rejected and tagged, a careful examination of the viscera was not made.

Several others of this herd approximating the condition of this steer were slaughtered immediately, and nearly all were condemned for the same cause. The remainder of the herd were allowed to rest for five days and they were fed on hay. In that time they again commenced to ruminate, the symptoms of weakness and nervous excitement or depression had disappeared. There remained only the lesions of the eyes—a permanent opacity of the cornea, varying from a small ulcer to a bulging staphyloma, discharging pus.

Post-mortem examination of the carcasses when made in the abattoir disclosed no pathological changes in the viscera. The liver, kidneys, and spleen were apparently normal in size, color, and consistency, but in some the fat adherent to the carcass was a disagreeable, deep-yellowish color, greasy to the touch, and in appearance resembling rancid butter.

On inquiry it was learned that many cattle are seen in the stockyards, very weak and poor in condition, and these cattle have an opacity of the cornea, and often a swelling of the legs. The history of these animals is always the same. While en route, or in confinement, they have been fed too much cotton-seed meal. The farmer and feeder, when questioned, says: "Yes, cotton-seed is a good feed for fattening cattle, but it often makes them go blind. Their eyes turn white."

I was unable to learn what particular preparation of the seed was fed to the cattle described. How long were they put on this feed? What quantity did they receive per head? and, Was cotton-seed used exclusively or with other food? are all questions that cannot be answered in this paper. They would certainly constitute important data that would throw much light on the condition described.

I believe that in many instances cotton-cake meal was fed exclusively. It was substituted for corn, and fed to the cattle in the cars. In some of the empty cars there were seen the cotton-seed hulls. It is probable that animals showing acute symptoms from being fed on cotton-seed exclusively while in confinement, and where the external fat is a dark-yellow color, that we hear the flavor of the beef is not relished, that it is inferior to corn-fed beef. This should be further demonstrated before accepted to be true of all animals fattened on cotton-seed. Fatty degeneration and infiltration of the tissues, with the excess of oily substance, doubtless occur to some extent. The swelling of the limbs, the continued venous pulse, and the hurried respiration, show that the action of the heart is very weak.

It appears difficult at first to account for the excessively high temperature in many of these animals. Weak action of the heart and depression of the vasomotor centres decrease the external circulation, and the heat of chemical change in the tissues, in excess because of the heat-producing cotton-seed aliment, which is not radiated from the surface, but confined in the interior tissues and membranes, may explain it.

Microörganisms that produce fevers, both specific and non-specific in character, are, doubtless, constantly entering the system, but if the body is in normal health the resisting vital forces render them passive or inert. When this vitality is lowered and the circulation is very weak toxin-producers immediately become active, and fever is soon apparent.

It is known that many animals fed on cotton-seed become blind. The acute symptoms are not often seen, as they are of brief duration, but it is believed that they are intimately connected with the lesions of the eye and are produced by the same cause.

An examination of the eyes from a number of these cattle showed abscesses of the cornea. These abscesses varied in size, from a minute point of opacity or small ulceration to a bulging staphyloma, occupying the entire area of the cornea and in many cases extending into and breaking down the adjacent interior tissues. From several there was a pustular discharge running down over the face and excoriating the skin. Where there was yet only a small opaque spot a closer examination disclosed a small ulcer containing small granules like dust or sand, appearing as foci. The position of this spot was generally in the centre of the cornea, at a line that would be made by the approximated lids. Where larger opaque areas were seen they were generally confined to the corneal surface. The smaller abscesses were well circumscribed. There was no inflammation of the surrounding conjunctiva of the eyelids, nor of the cornea and the membrana nictitans. No vascular zone of distended bloodvessels at the junction of the cornea and sclera was apparent. In some animals only one eye was affected. This was sufficient to eliminate catarrhal conjunctivitis. Where the staphyloma occupied all the areas of the cornea and bulged from the normal surface in the form of a blister or bladder it acted as a mechanical obstruction to the closing of the lids. The superior lid was retracted and appeared as a swelling over the uncovered opaque cornea, and this caused a very painful expression. A number of these eyes were dissected, and the optic nerve and adjacent anterior tissues were found to be normal in size and appearance where the abscesses were confined to the cornea.

It is known that repeated irritation of the digestive tract will cause serious derangement to the nervous system. That it may ultimately result in affecting the cells of the cornea and their nutrition is not improbable. The cells of the cornea tissue proper contain very many terminal nerve-filaments, but it is very poorly supplied with bloodvessels, and therefore predisposed to loss of sensation. Sensation of the cornea, and particularly the conjunctival layer, and the membrana nictitans is supplied by the terminal nerve-filaments of the ophthalmic branch of the fifth pair of cranial nerves, or the trigemini, through its sensitive roots, and in the conjunctiva there are also the corpuscles of Krouse. In the healthy animal, when these tissues are irritated, the secretion of the lachrymal glands, stimulated by reflex action, washes off particles and foreign bodies and lubricates the corneal surface. By this process of "tearing" traumatic disturbances are prevented that would be caused when dust and sand are blown against the surface.

In severe depression of the central nervous system the functional activity is decreased, and in extreme conditions, as paralysis, there is an absence of tears as a result of complete anæsthesia of the conjunctiva, especially of the corneal surface. The cornea, then, appears dry and loses its gloss. When the cornea becomes anæsthetized through any cause, as paralysis of the fifth nerve, dust and foreign particles lodge in the conjunctival surface, lachrymation does not appear to remove, and they become the foci for abscess-formation. By extension of the irritation the entire substance of the cornea becomes organized and appears opaque. At first there is seen a small ulcer; pyogenic bacteria enter these minute abrasions; soon the cornea begins to suppurate, and a staphyloma appears. When the abscess once starts the cornea never recovers its normal transparency. Sensation of the cornea may be only temporarily lost, yet the effect of traumatisms may be sufficient to cause the entrance of microörganisms. When the suppuration starts it continues unchecked even if sensation returns.

Continued irritation of the cotton-seed aliment to the mesenteric nerve lining the surface of the intestines is conducted through the sympathetic chain to the spinal cord. By reflex action through the cardio-inhibitory centres of the medulla, and by the pneumogastric nerve, the action of the heart is inhibited both in force and frequency and the cardiac muscles are relaxed. It has been proved that the continuous irritation to the sympathetic nerve will also inhibit the vasomotor centres in the medulla and cause a decrease in arterial pressure. Arrest of the circulation and slowing of the blood-current along the paths of the cranial nerves, as the trigeminus, and blood stasis in the capillaries at their terminal filaments, may result in a loss of sensation, for sensation is dependent on a high degree of vascularity.

The following description is quoted from the works of two authorities on the diseases of the eye, and the lesions and conditions are similar to those seen in these cattle:

"Neuro-paralytic keratitis—an ulcerated condition of the cornea

—arising when the structure becomes anæsthetic, because it is severed from the influence of the trigemini. Cause: The corneal lesion has been ascribed to a trophic change, to the lessened power of resistance to microörganisms, which the cornea in its insensitive condition presents to external injury, to the irritation to the fifth nerves, and to increased evaporation from the surface of the cornea; disease of the Gasserian ganglion; disease of the nuclei of the fifth pair; anything which cuts off trigeminal influence; foreign substances remaining undetected upon the insensitive cornea, whose resisting power is weakened through loss of trophic influence. Symptoms: The corneal tissue is comparatively clear beyond and around the central abscess. In periphery there are secondary foci of infiltration closely connected with the inflammation of the neighboring conjunctiva. The surface of the cornea and the conjunctiva is anæsthetic. There may be pain and irritation or there may be abscess."—De Schweinitz (*Diseases of the Eye*, second edition, p. 280).

"When the fifth pair and particularly the part containing fibres of the sympathetic is divided in animals, in a short time the cornea on that side begins to ulcerate and soon passes on to total destruction. Also, when the fifth pair is from any cause paralyzed in man, and particularly when the branch going to the orbicularis muscle is involved at the same time. It is a point in dispute, whether the ulceration is due to interference with nutrition from injury to the trophic filaments of the fifth pair, or is simply the result of traumatic injuries inflicted on the insensitive cornea on account of its constant exposure from paralysis of the orbicularis. Injury to the trophic nerves seriously impairs the resisting power of the corneal tissue, and in some instances is itself sufficient to bring about destructive inflammation."—S. M. Burnett, Washington, D. C.: *American Text-book of Diseases—Diseases of the Eye, Ear, Nose, and Throat; Diseases of the Cornea and Sclera.*

In this paper it was not intended to detract from the value of cotton-seed as a food.

From the number of cattle that have arrived at the stock-yards from the South, weak and blind and suffering with acute symptoms, it would appear that many feeders are not familiar with deleterious results from injudicious feeding, and in conclusion a short review of what has been learned by experiment may not seem superfluous here.

The best material has been found to be the cakes made from the pressed hulled seeds. A large part of the hull is a comparatively indigestible substance, and they should never be fed exclusively as a substitute for hay. Cattle should always be gradually accustomed to this food, and in any form it should never be fed alone, but always dry and mixed with other forms of fodder.

*Discussion.*

DR. STEWART: It was rather unfortunate that I was appointed to open the discussion on this paper, for the reason that I have not had opportunity to acquire a definite knowledge of the disease as it appears in animals in this section, neither have I had any experience in treating it. I am very much interested in the paper, however, and have seen two or three bunches of cattle suffering from what was said to be overfeeding with cotton-seed meal.

The paper laid very considerable stress upon the lesions found in the eyes, and I must say the eye lesions are quite prominent and sure to attract attention. The external lesions are similar to those found in contagious ophthalmia of cattle, with this difference, that the opacities in contagious ophthalmia are circular and centrally located on the cornea and inclosed with an inflammatory ring or zone, while in the disease produced by cotton-seed the opacities are centrally located but elongated in the direction of the margin of the eyelids and do not have an acute inflammatory zone surrounding them. My observations differ somewhat from that of the authors in that in many cases which I saw there was considerable tearing, the tears producing wet streaks down the face, commencing at the inner canthus of each eye.

It is generally understood in the section where cotton-seed is largely used that it is one of the best agents with which to fatten cattle, but intelligent feeders are aware that if fed too long the cattle will cease to lay on fat, and that serious disturbances of health will develop. If memory serves me correctly, Dr. Cary wrote an article several years ago relative to the value of cotton-seed as food for swine, in which he made the statement that if swine were fed too long on it there would develop a form of scurvy as well as other constitutional disorders.

DR. BENNETT: In this connection I would say that some weeks ago I observed about twenty-three head of cattle that came into Kansas City stock-yards. Upon investigation I learned that these cattle came from below the quarantine line, and had been fed on cotton-seed about one hundred and ten days, and they were so badly affected that we deemed it necessary to hold them for post-mortem examination. In the meantime two of them had died in the stock-yards, and upon taking the temperature of the remaining twenty-one we found that they varied from 103° to 108° F. I also observed this peculiar condition of the eyes some speak about. Some seemed to be entirely blind, and most of them had a discharge from the eye that ran down over the face. Some of the cattle had a staggering gait and swelling of the legs, about the same as you observe in a horse after standing in a stable for a time. The examination was continued next day, when the cattle were killed and we found lesions that resembled Texas fever very much. While we did not find characteristic lesions of Texas fever we found in some instances that the spleen was very much enlarged.

I have learned from various feeders that have used cotton-seed that from seventy to eighty days is about the proper length of time to feed cattle, as after this time they seem to lose ground, and it is advisable to get them into the market as soon as possible.

As far as I am able to learn no investigations have been made, with the exception of a few feeding experiments on some calves. Very soon after the above cases I had an opportunity to make a post-mortem examination of two steers that had been fed on cotton-seed for about ninety days. They had taken on fat very rapidly, and had become sick very suddenly. They were shipped to Kansas City, where they were killed, but not in the usual manner by knocking them in the head, but stuck them alive. We found enlarged spleens in these two cattle, also enlarged livers; the bile was slightly changed from the normal and the urine was highly colored. This is something I think ought to be investigated, as we see it quite frequently in Kansas City among cattle that have been fed on cotton seed and the animals lose their sight, and we find the internal organs presenting abnormal appearances.

Dr. Wilson: In these twenty-three head of cattle how many did you condemn?

Dr. Bennett: Seven out of twenty-three. It is claimed by some old feeders that they can tell a cotton-seed steak as soon as they see it. The cattle we had there were not bright at all, and the seven we had condemned showed the highest temperature. Three of the seven showed marked change in the liver, and resembled Texas fever to some extent. In one case we found something similar to a hobnailed liver.

